# Research protocol: Cervical Arthroplasty Cost Effectiveness Study (CACES): economic evaluation of anterior cervical discectomy with arthroplasty (ACDA) versus anterior cervical discectomy with fusion (ACDF) in the surgical treatment of cervical degenerative disc disease — a randomized controlled trial

**DOI:** 10.1186/s13063-022-06574-5

**Published:** 2022-08-26

**Authors:** Valérie N. E. Schuermans, Anouk Y. J. M. Smeets, Toon F. M. Boselie, Math J. J. M. Candel, Inez Curfs, Silvia M. A. A. Evers, Henk Van Santbrink

**Affiliations:** 1 Department of Neurosurgery, Maastricht University Medical Center+, P. Debyelaan 25, Maastricht, 6229 HX The Netherlands; 2 Department of Neurosurgery, Zuyderland Medical Center, Henri Dunantstraat 5, Heerlen, 6419 PC The Netherlands; 3 CAPHRI School for Public Health and Primary Care, Maastricht University, Universiteitssingel 40, Maastricht, 6229 ER The Netherlands; 4 Department of Methodology and Statistics, Care and Public Health Research Institute (CAPHRI), Maastricht University, Peter Debyeplein 1, Maastricht, 6229 HA The Netherlands; 5 Department of Orthopaedic Surgery and Traumatology, Zuyderland Medical Center, Henri Dunantstraat 5, Heerlen, 6419 PC The Netherlands; 6 Department of Public Health Technology Assessment, Maastricht University, Duboisdomein 30, Maastricht, 6229 GT The Netherlands; 7 Trimbos Institute, Netherlands Institute of Mental Health and Addiction, Centre of Economic Evaluation & Machine Learning, Utrecht, The Netherlands

**Keywords:** Cervical degenerative disc disease, Cervical myelopathy, Cervical radiculopathy, Anterior cervical discectomy and fusion, Anterior cervical discectomy with arthroplasty, Cost-effectiveness

## Abstract

**Introduction:**

To date, there is no consensus on which anterior surgical technique is more cost-effective in treating cervical degenerative disc disease (CDDD). The most commonly used surgical treatment for patients with single- or multi-level symptomatic CDDD is anterior cervical discectomy with fusion (ACDF). However, new complaints of radiculopathy and/or myelopathy commonly develop at adjacent levels, also known as clinical adjacent segment pathology (CASP). The extent to which kinematics, surgery-induced fusion, natural history, and progression of disease play a role in the development of CASP remains unclear. Anterior cervical discectomy with arthroplasty (ACDA) is another treatment option that is thought to reduce the incidence of CASP by preserving motion in the operated segment. While ACDA is often discouraged, as the implant costs are higher while the clinical outcomes are similar to ACDF, preventing CASP might be a reason for ACDA to be a more cost-effective technique in the long term.

**Methods and analysis:**

In this randomized controlled trial, patients will be randomized to receive ACDF or ACDA in a 1:1 ratio. Adult patients with single- or multi-level CDDD and symptoms of radiculopathy and/or myelopathy will be included. The primary outcome is cost-effectiveness and cost-utility of both techniques from a healthcare and societal perspective. Secondary objectives are the differences in clinical and radiological outcomes between the two techniques, as well as the qualitative process surrounding anterior decompression surgery. All outcomes will be measured at baseline and every 6 months until 4 years post-surgery.

**Discussion:**

High-quality evidence regarding the cost-effectiveness of both ACDA and ACDF is lacking; to date, there are no prospective trials from a societal perspective. Considering the aging of the population and the rising healthcare costs, there is an urgent need for a solid clinical cost-effectiveness trial addressing this question.

**Trial registration:**

ClinicalTrials.gov NCT04623593. Registered on 29 September 2020.

**Supplementary Information:**

The online version contains supplementary material available at 10.1186/s13063-022-06574-5.

## Article summary

### Strengths and limitations of this study

- External validity and generalizability are limited, as costs are country-specific.
- A broad economic analysis from a societal perspective in the setting of a prospective randomized controlled trial will investigate the cost-effectiveness of ACDA and ACDF for CDDD, resulting in level I evidence.
- The sample size is based on estimated costs and average yearly incidence of CASP, which may differ from reality.
- The burden for patients in this study is low, and potential risks in the intervention group (ACDA) are similar to those in the control group (ACDF with stand-alone cages), which is standard care in our center.
- Since the gap in incidence of additional CASP-related surgery between ACDF and ACDA expands considerably with time, we expect that a difference in costs, if found within the 4-year follow-up period, will also increase over time.

## Introduction

Cervical degenerative disc disease (CDDD) is the degeneration of a cervical intervertebral disc and/or the adjoining vertebral bodies, resulting in clinical symptoms of cervical radiculopathy, myelopathy, myeloradiculopathy, and axial pain. The incidence of degenerative pathologies is significantly increasing as the proportion of elderly in the population is rising [1, 2]. Currently, generalized spinal disc degeneration occurs in more than 90% of adults over 50 years old [3]. This age group now represents 32.8% of the population in Europe and is projected to reach 40.6% by 2050 [2]. In the next 20 years, a significant increase in anterior cervical decompression surgeries is predicted in people aged 45–54, mainly affecting the working population [4–6]. Symptoms of radiculopathy and/or myelopathy lead to restrictions in daily life and loss of professional capability, resulting in absenteeism. Societal healthcare costs are therefore significantly affected by CDDD. Further costs of healthcare are accrued when patients require surgical treatment, combined with associated hospitalization and rehabilitation. Currently, there are several (surgical) treatments for CDDD available. To date, there is no consensus on which surgical technique is more cost-effective in treating CDDD with radiculopathy and/or myelopathy.

One of the most common procedures for treating patients with single- or multi-level CDDD is anterior cervical discectomy with fusion (ACDF) [7, 8], which results in fusion in 95–100% of the cases [9]. Axial pain alone is not considered an indication for surgical treatment in our country. The primary goal of ACD(F) is the relief of symptoms of radiculopathy and/or myelopathy through decompression of neural structures. Fusion in itself is not a requisite to reach this goal. In our center, ACDF with stand-alone cages is the standard procedure for CDDD. Plate constructs are only used in case there are signs of instability (e.g., spondylolisthesis) and additional stabilization is deemed necessary. A common concern regarding ACDF with stand-alone cages is the occurrence of cage subsidence. However, in our recent retrospective cohort of 673 patients, only 1 patient required additional surgery due to subsidence (0.15%) [10]. Good short-term clinical results are achieved for both radiculopathy and myelopathy [9, 11]. Clinical results are independent from the technique used and from the occurrence of fusion [9, 11–13]. However, patient-reported satisfaction gradually decreases in the years following surgery [14, 15]. This is thought to be the consequence of the development of new complaints due to degenerative changes at a segment adjacent to the site of the index surgery, also known as adjacent segment pathology (ASP) [16].

A recent consensus proposes a distinct definition that distinguishes between *radiologic* adjacent segment pathology (RASP) and *clinical* adjacent segment pathology (CASP) [16]. CASP occurs at an estimated cumulative rate of 1.6–4.2% per year after ACDF [16, 17]; however, the incidence reported in literature varies widely [18–21]. Nevertheless, 50–75% of the patients that develop CASP require additional adjacent segment surgery [17, 22–27]. In our retrospective cohort, we observed an average rate of 2.1% of CASP per year, with an additional adjacent segment surgery rate at an average of 1.5% per year [10]. The annual incidence was unevenly distributed as half of these additional surgeries for CASP occurred within 2.5 years, which suggests a peak incidence in the first years following the index surgery [10].

The underlying mechanism of ASP remains a matter of debate. Besides natural progression of degeneration, compensation for the loss of motion in the fused segment is thought to cause overstraining of the adjacent segments [28–30]. Altered cervical sagittal alignment is also thought to be important in the accelerated development of CASP. Indeed, higher rates of CASP are observed after ACD, concomitant with an increased segmental kyphosis at the index level [12, 31, 32]. Unlike ACD, ACDF with plate constructs restores cervical sagittal lordosis. However, a higher rate of ASP is observed in patients with plate constructs compared to ACDF with stand-alone cages [12, 33]. This finding might be explained by the plate causing strain on the adjacent segments, or the more extensive surgical preparation for installing the plate, which increases the chance of iatrogenic damage to the adjacent level. Another contributing factor might be the occurrence of subsidence of the plate construct into the adjacent segment. Disc height at the adjacent segments has been found to be significantly decreased in patients with plate constructs, which supports this theory [33]. Nevertheless, the extent to which altered cervical motion influences the development of ASP remains unknown [34, 35].

Anterior cervical discectomy with arthroplasty (ACDA) was developed as another treatment option for cervical radiculopathy and/or myelopathy to reduce the incidence of CASP by preserving motion in the operated segment. Previously conducted research in patients with radiculopathy and/or myelopathy has shown that clinical and radiological outcomes are similar between ACDA and ACD(F) [28, 36, 37]. A meta-analysis found better neurological outcomes in patients with myelopathy after ACDA, in contrast to the pre-existing notion that ACDA leads to less favorable outcomes in myelopathy due to micro-trauma caused by preserved mobility [38]. Moreover, additional adjacent segment surgery rates are significantly lower for ACDA, for single- and multi-level surgeries [39, 40]. The difference in additional adjacent segment surgery rates between ACDA and ACDF increases exponentially with longer follow-up time (Table 1). Despite these long-term benefits of ACDA, it is often discouraged since the clinical outcomes are similar to ACDF, but the implant costs are higher. However, preventing new complaints and additional surgeries due to CASP might be a reason for ACDA to be a more cost-effective technique in the long term. A systematic review of economic evaluations in anterior cervical decompression surgery was conducted by our research group [41]. Indeed, the majority of studies reported ACDA to be the most cost-effective technique despite the higher implant costs. However, literature was highly heterogeneous and of low quality. Hence, although there is increasing evidence suggesting that ACDA might be the more long-term cost-effective technique because of the reduced risk on CASP and associated additional surgery rates compared to ACD(F), the quality of this evidence is lacking, especially in Europe. Therefore, the need for a solid clinical cost-effectiveness trial addressing this question is high. Currently, ACDA cannot be offered as a standard treatment in the Netherlands because it is not reimbursed as the costs for ACDA are higher and short-term symptom relief appears equal between the techniques.

**Table 1:** Incidence rates of additional surgery for CASP

	FU time (years)	ACDA (%)	ACDF (%)	Annual rate ACDA (%)	Annual rate ACDF (%)
**Trial registration NCT00389597**					
Radcliff [42]	7	3.7 4.4	13.6 11.3	0.5	1.9
Radcliff [39]	5	3.1	11.4	0.6	2.3
Jackson [43]	5	4.5 7.3	17.3 21	0.9 1.5	3.5 4.2
Radcliff [44]	3	5.7	10.5	1.9	3.5
Davis [45]	2	0.9	0	0.4	0
**Trial registration NCT0029108**					
Loumeau [46]	7	0	18.2	0	2.6
Janssen [47]	7	7	18	1	2.6
Zigler [48] and Delamarter [49]	5	2.9	11.3	0.6	2.3
Murrey [50]	2	1.8	8.5	0.9	4.2
**Trial registration NCT00637156**					
Gornet [51]	10	9	17.9	0.9	1.8
Gornet [52]	7	11.6 6.5	10.9 12.5	0.9	1.8
Lanman [53]	7	6.5	12.5	0.9	1.8
Gornet [54]	2	2.4	3.2	1.2	1.6
**Trial registration NCT642876**					
Burkus [55]	7	4.6	11.9	0.7	1.7
Mummameni [56]	2	1.1	3.4	0.5	1.7
**Trial registration NCT00437190**					
Loidolt [57]	10	9.7	15.8	1.0	1.6
Lavelle [58]	10	9.7	15.8	1.0	1.6
Sasso [59]	4	4.1	4.1	2.8	1.7
Heller [60]	2	2.5	3.6	1.2	1.8
**Average [range]**				**1.0 [0; 2.8]**	**2.2 [0; 4.2]**

Equipoise remains concerning the cost-effectiveness of these techniques; the main aim of CACES is to determine the long-term cost-effectiveness of ACDA in comparison to ACDF. As the difference in re-operation rates for CASP is expected to be lower after ACDA, we hypothesize that the initial higher costs of a disc prosthesis might reimburse itself on the long term. The study aims to investigate a population that reflects daily practice; therefore, both radiculopathy and myelopathy and single- and multi-level patients will be included.

## Methods

### Objectives

The primary objective of the proposed study is to examine whether ACDA is preferred over ACDF in terms of cost-effectiveness from a healthcare and societal perspective, in patients with single- or multi-level symptomatic CDDD. Secondary objectives include differences in clinical and radiological outcomes between the two techniques, as well as the qualitative process surrounding anterior decompression surgery.

### Study design

This is a prospective, single-blinded, randomized controlled trial. One study arm receives ACDA (intervention group), and the other receives ACDF (control group). Patients will be randomized in a computer-generated 1:1 ratio to ensure equal division among both groups. No block randomization or stratification will be used. Only the coordinating investigator will be able to perform the computer-generated randomization. Group allocation will be performed per patient, not per level, meaning that no hybrid constructs will be used in this study. CACES investigators and surgeons will not be blinded for treatment allocation. All other involved healthcare professionals are blinded for allocation. All visible documentation will report concerning “ACD,” unspecified whether it concerns fusion or a disc prosthesis. A follow-up period of 4 years was chosen to encompass the expected incidence of 1.6–4.2% of additional CASP-related surgeries of per year, with a peak incidence in the first 2.5 years [16]. It is reasonable to expect that most CASP events will occur within this follow-up period and that we will be able to gather reliable cost data of patients and informal caregivers (ICGs).

### Participants and recruitment

Patients will be enrolled in the Department of Neurosurgery, Zuyderland Medical Center, Heerlen, the Netherlands. All adult patients indicated for single- or multi-level surgery for radiculopathy and/or myelopathy due to CDDD are eligible to be included in the study.

If patients choose not to participate in the study, the standard treatment, ACDF with stand-alone cage, will be offered. If patients agree to be included in the study, they will complete the informed consent form. ICGs will also be asked to participate, to enable a broad societal evaluation of costs. Patients can leave the study at any time for any reason without consequences. The investigator can decide to withdraw a patient from the study for urgent medical reasons. Since the sample size accounts for a 20% loss to follow-up, patients will not be replaced after withdrawal.

### Inclusion criteria

In order to be eligible to participate in this study, a patient must meet all of the following criteria:

- Indication for anterior cervical decompression surgery
- Single- or multi-level CDDD, located between C3 and C7, with a maximum of 4 consecutive levels
- Symptoms of myelopathy, radiculopathy, or myeloradiculopathy
- In the case of pure radiculopathy: refractory to at least 6 weeks of conservative therapy
- In the case of myelopathy, it must be symptomatic
- Patients ≥18 years of age

### Exclusion criteria

Patients who meet any of the following criteria will be excluded from participation in this study:

- Indication for (additional) posterior surgical approach
- Indication for additional stabilization of the pathological segment by a plate; in case there are sign of instability (e.g., spondylolisthesis) and additional stabilization is deemed necessary
- Previous surgery of the cervical spine (anterior and posterior)
- Traumatic origin of the compression
- Previous radiotherapy of the cervical spine
- Metabolic bone disease
- Inflammatory spinal disease, e.g., Bechterew’s disease, Forestier’s disease
- Infection of the cervical spine
- Unable to fill out Dutch questionnaires
- Informed consent not possible

Dropout criteria include technical reasons related to placing either of the implants during the surgery. Other reasons for dropout are expected to be low.

### Interventions

One of two surgical procedures will be performed on patients participating in this study: ACDA (intervention group) and ACDF (control group). In both techniques, the disc space is approached through a right- or left-sided anterior surgical approach. The disc content will be removed, after which the endplates are prepared with curettes. The posterior longitudinal ligament is opened, and the dura is visualized to ensure adequate decompression. In the ACDF group, a cage (CeSPACE® XP) is implanted; in the ACDA group, a disc prosthesis (ActivC®) is implanted after the discectomy. No bone substitutes will be used.

Implantation of the devices is done in accordance with the manufacturer’s protocol for implantation and endplate preparation. In both groups, the wound is closed in layers, after a prevertebral wound drain is placed. Potential surgical risks are similar between the cage (ACDF) and the arthroplasty group (ACDA) [38, 61].

Post-surgical care is the same for both groups. Patients receive 24 h of antibiotic prophylaxis, and after 24 h, the prevertebral wound drain is removed. Patients can use analgesics post-operatively, and they are allowed to mobilize immediately. The day after surgery, a physical therapist provides patients with standardized exercises. Patients are not routinely referred to a physical therapist, and no neck collar is used.

### Implementation plan

**Fig. 1:**
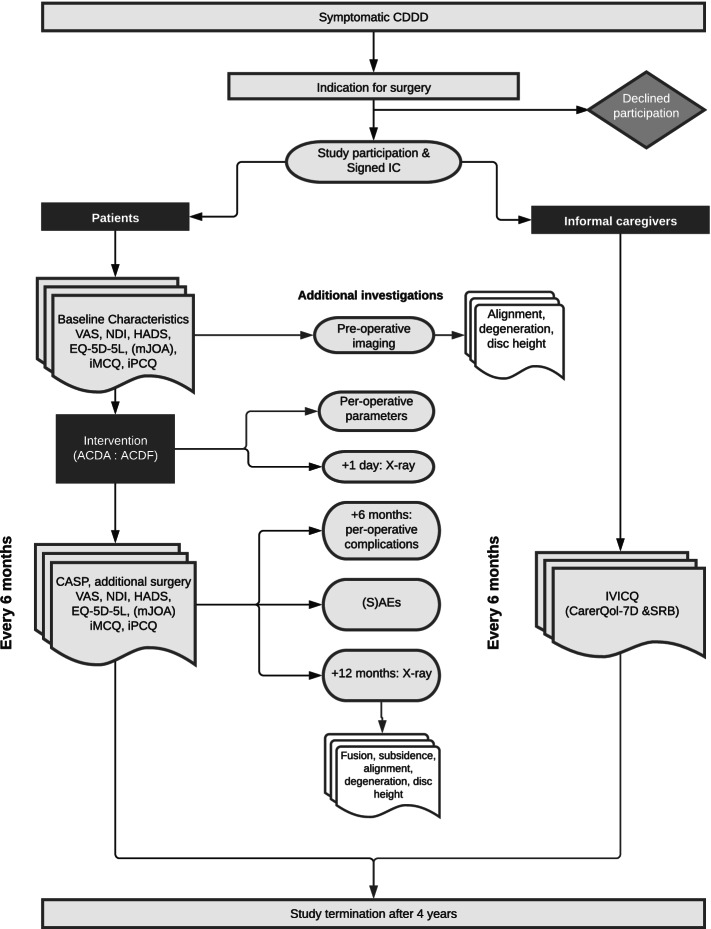
Study design. Timing of pre- and post-operative outcome measurements and follow-up evaluations

Figure 1 depicts the flowchart of the study process. Patients will be selected by screening the appointments in the neurosurgical outpatient clinic the waiting lists for surgery. When a patient is indicated to undergo anterior cervical decompression surgery, the treating neurosurgeon will inform the patient about the CACES trial. The patient is contacted by the investigator to provide extensive information. Additionally, written information is sent to them by (e)mail. A week after the information is sent, the investigator contacts the patient again to answer remaining questions. Patients are included in the study when informed consent is signed. Data is collected in the research manager tool, which is a database with a built-in auditing system. Questionnaires, including reminders, are sent automatically once the patient is registered in Research Manager. If patients or informal caregivers prefer to fill out questionnaires on paper, the answers will be registered in Research Manager by the investigator and the paper version will be stored. Data will be collected at baseline and at 6-month intervals until 4 years post-surgery. The patients will be blinded to their received treatment for the first year after surgery to obtain more reliable patient-reported outcome measures (PROMs). After 1 year, patients will have an outpatient appointment with the coordinating investigator for unblinding (if wanted) and additional imaging.

### Outcome measures

All outcomes will be measured at baseline and at 6-month intervals until 4 years post-surgery.

#### Primary outcome

The primary outcome is cost-effectiveness and cost-utility of ACDA compared to ACDF from a societal perspective, in patients with single- or multi-level CDDD and symptoms of radiculopathy and/or myelopathy. The primary outcome will be evaluated at 4 years post-operative.

The economic evaluation will involve a combination of a cost-effectiveness analysis (CEA) and cost-utility analysis (CUA) from a societal perspective. In the CUA, the effects are presented as quality-adjusted life years (QALYs), based on the EuroQol 5 dimensions 5 levels (EQ-5D-5L) [62] utility scores [63]. The Net Monetary Benefit (NMB) will be determined, in which effect is expressed in QALYs. The incremental cost-effectiveness ratio (ICER) will be expressed as the incremental costs per QALY. This economic evaluation will be conducted according to the Dutch Guidelines of the National Health Care Institute [64–66]. The following validated cost questionnaires will be used: Productivity Cost Questionnaire (iPCQ) [67] and the Medical Consumption Questionnaire (iMCQ) [67]. These questionnaires allow a broad measurement of societal costs, including medical consumption, and both paid and unpaid loss of productivity for patients. ICGs will be asked to fill out the Limited Valuation of Informal Care Questionnaire (iVICQ) [68], which consists of the Care-related Quality of Life instrument (CarerQol-7D) and the Self-Rated Burden scale (SRB). This measures the (subjective) burden for ICG and the assessment of caregiving in terms of wellbeing.

#### Secondary outcomes

Secondary outcomes will consist of both clinical and radiological outcome measurements.

Clinical outcomes will be assessed according to the rate of CASP and associated additional surgeries, combined with patient-reported outcome measures (PROMs). The included PROMs are the Neck Disability Index (NDI) [69], Visual Analogue Scale (VAS) [70] for neck and arm pain, Hospital Anxiety Depression Scale (HADS) [71], and the modified Japanese Orthopedic Association score (mJOA) [72] for myelopathy or myeloradiculopathy patients. CASP is defined as the presence of newly developed symptomatic cervical radiculopathy and/or myelopathy on a level adjacent to the initial surgery, confirmed by corresponding findings upon magnetic resonance imaging (MRI). It should be noted that *RASP* is differentiated from *CASP* by the presence of clinical symptoms that are attributed to the degenerative changes for the latter. Patients were only considered as having CASP when new clinical symptoms developed. Additional adjacent segment surgery for CASP was defined as surgery for radiculopathy and/or myelopathy at a segment adjacent to the level of initial surgery. Notably, neck pain itself is not considered as a surgical indication in our national guidelines [73]. Re-operations at the index level and at levels not adjacent to the initially operated level were not considered as additional surgery for CASP.

Radiological outcomes will be assessed at three time points: pre-operative, directly post-operative, and 1 year post-operative. Pre-operative imaging will assess baseline degeneration according to the Kellgren-Lawrence Score (KS) [74], cervical sagittal alignment, and baseline disc height. Moreover, a full sagittal spine X-ray will be taken to assess pre-operative global balance according to the odontoid-hip axis (OD-HA) [75]. A standard cervical spine X-ray will be taken at the immediate post-operative period (before discharge) to assess the position of the implant, subsidence, and cervical sagittal alignment. Cervical spine X-rays will be taken again 1 year post-operative to assess fusion, cage subsidence, adjacent segment degeneration (KS), adjacent segment disc height, and alignment. A flexion and extension X-ray will be taken to assess movement. Radiological cage subsidence will be defined as > 3-mm decrease of interbody height compared to the direct post-operative X-ray [76, 77].

Moreover, a process evaluation will be performed to determine the underlying values, needs, impacts, and preferences of people with CDDD. The focus will be on the experiences and opinions of patients, caregivers, and professionals concerning the process surrounding ACDA and ACDF. A process evaluation might also identify gaps or limitations in the published research with regard to important outcomes to those with lived experience. A qualitative analysis will be performed according to the framework provided by Saunders et al. [78].

#### Covariates

Per-operative parameters such as intraoperative complications, operative time, size of the implant, and blood loss will be evaluated. Perioperative complications, re-operations at index- or non-adjacent levels, and their associated costs will be assessed.

### Power considerations

The primary outcome of the proposed study is cost-effectiveness. No difference in QALYs is expected between the intervention and control groups based on previous literature [41].

The only expected difference in clinical effectivity is the additional adjacent segment surgery rate, with an average of 1.0% versus 2.2% (Table 1). Although small, a difference in additional surgery rates is present in all trials, increasing exponentially over the years. We believe that the small inter-group difference may result in a cost-effective intervention, mainly because we expect most of the difference in costs to arise from episodes of secondary symptoms, which can lead to absenteeism, use of analgesic medication, use of (para)medical supplies, and additional adjacent segment surgery.

Reported additional adjacent segment surgery rates for ACDA vary from 0 to 2.8% per year, with an average of 1.0% based on the largest FDA/IDE trials [46, 79]. As for ACDF, yearly reported additional adjacent segment surgery rates range from 0 to 4.2%, with an average of 2.2% [80, 81].

The cost of a cervical disc prosthesis for ACDA is estimated to be around €2000, while the cage for an ACDF costs €500, resulting in a difference of €1500 per implant. Based on our data from the past years, on average, 1.2 implants are used per procedure.

Hospital admission costs and procedural costs are similar for both procedures, which is estimated to be €11,000 in our center. As reported by ArboNed for the year 2019, the average absenteeism for cervical disc pathology was 242 days, with a standard deviation of 225 *[personal communication]* [82]. The average cost for 1 day of absenteeism varies between €200 and €400, depending on salary and company; for example, 1 day of absence from work is usually calculated to cost around €250 in the Netherlands *[personal communication]* [82].

In the Netherlands, the willingness to pay (WTP) threshold for spinal pathologies — classified as a moderate disease — is set at €50,000/QALY [65, 66]. However, in the sample size calculation, we will not fix WTP at one value. The sample size calculation is based on both QALYs and costs, using Net Monetary Benefit (NMB) as outcome. The NMB for a treatment is defined as [83]:

$$NMB= WTP\times Effect- Costs$$

The smallest relevant effect in terms of NMB will be quantified in terms of Cohen’s *d*, which is defined as:

$$d=\frac{average\ NMB\ (ACDF)- average\ NMB\ \left(\mathrm{ACDA}\right)}{Standard\ deviation\ of\ NMB}$$

The standard classification of Cohen’s *d* consists of a small effect (*d* = 0.2), a medium-sized effect (*d* = 0.5), and a large effect (*d* = 0.8) [84].

A two-sided test will be used with a 5% level of significance (*α* = 0.05). A Cohen’s *d* of 0.4 is often referred to as the hinge point, meaning a greater than average effect [84]. We expect a bit less than a medium-sized effect, for which we choose a Cohen’s *d* in between 0.5 and 0.4, resulting in a Cohen’s *d* of 0.45. Inclusion of 79 patients per treatment group will then yield a power of 0.80. Taking into account a 20% loss to follow-up in 4 years, the required sample size is 99 per group, with a total of 198 patients.

### Statistical analysis

All statistical procedures will be conducted based on both the intention-to-treat principle and on actual participation in treatment (i.e., per-protocol analyses) and will be performed using SPSS statistics (SPSS, IBM, Corporation, Chicago, USA). Effect sizes and their 95% confidence intervals will be estimated. A two-sided significance level of 0.05 will be used as a threshold to determine whether differences are statistically significant. Trends in differences between ACDA and ACDF will be measured over time. If significant differences are found, post hoc analyses further investigate these effects.

Costs will be calculated by multiplying volumes (resource use) with unit costs. For the unit costs, we will use the Dutch costing guidelines. Productivity costs will be calculated by means of the friction cost method, based on a mean added value of the Dutch working population. Cost prices (2022) will be expressed in euros. If necessary, existing cost prices will be updated using the consumer price index (2022).

### Patient and public involvement

This study is supported by the national Dutch patient society of spine patients (‘Nederlandse Vereniging van rugpatienten ‘de Wervelkolom’) as well as by the Dutch national society of neurosurgeons (NVVN).

### Data management

To protect the privacy of all participants, all collected data will be encoded. Data collection will be carried out using the data management module from the research manager at Zuyderland. This application for detailed custom-made electronic data collection meets all guidelines of Good Clinical Practice (GCP). Trial auditing is incorporated in this system. Handling of personal data will comply with the General Data Protection Regulation (GDPR) (in Dutch: Algemene Verorderning Gegevensbescherming (AVG)). Informed consent forms and questionnaires filled-out on paper will be stored for 15 years in a safe location. Only the coordinating investigators will have access to the encoding key. ICG information will automatically be linked to the specific patient they are taking care of. During the study, the encoded data will be accessible on request. After the study, the encoded data will be publicly accessible for verification or further research. Monitoring will be performed by trained and qualified monitors from an external party, remotely and on site (Clinical Trial Centre Maastricht). All (serious) adverse events will be recorded. All patients are insured for participation in this trial and the possible consequences.

### Protocol amendments

All substantial amendments will be notified to the METC and to the competent authority. A “substantial amendment” is defined as an amendment that is likely to affect the safety or physical or mental integrity of the subjects of the trial, the scientific value of the trial, the conduct or management of the trial, or the quality or safety of any intervention used in the trial. Non-substantial amendments will not be notified to the accredited METC and the competent authority, but will be recorded and filed.

### Ethical principles

This research will be conducted according to principles enshrined in the Declaration of Helsinki (3rd edition 2013) and in accordance with the Dutch Medical Research Involving Human Subjects Act (WMO). The study protocol has been approved by the Medical Ethical Committee of the Zuyderland Medical Center, Heerlen (document nr: METCZ20200025, NL72534.096.20). Written informed consent will be obtained from all patients included in the study.

Patients are insured and covered for damage to research subjects through injury or death caused by the study. The insurance applies to the damage that becomes apparent during the study or within 4 years after the end of the study.

## Discussion

There is currently no consensus on which anterior surgical technique is most cost-effective for treating CDDD. The role of ACDA in the prevention of ASP remains a controversial topic, which is a consequence of the ambiguous accelerating factors of ASP. Current literature reports inconsistent incidence rates and controversial risk factors. The extent to which kinematics, surgery-induced fusion, and natural history of disease play a role in the development of ASP remains unclear. Therefore, this study aims to provide high-quality evidence concerning the cost-effectiveness of ACDA in comparison to ACDF, as well as to evaluate the proposed accelerating factors of ASP. This is of dual importance; on the one hand, it will provide optimal patient care, and on the other hand, it will limit the exponentially increasing healthcare costs.

Based on the available literature, a difference in clinical effectiveness between the two treatments in the short term (1 to 2 years after surgery) is not expected [35, 85–89], as the risk profiles for ACDF and ACDA surgeries are comparable [61]. Aside from the high implant costs, ACDA is sometimes discouraged because of the concern that it leads to less favorable outcomes in myelopathy [61, 90, 91]. However, current literature provides no evidence supporting this concern and even reports better neurological outcomes after ACDA [61, 90–92]. Moreover, revision rates at the index level appear to be similar for ACDA and ACDF. As such, in our opinion, ACDA poses no additional risk for patients [93].

A concern that may arise regarding this study protocol is the use of ACDF with stand-alone cages instead of plate constructs, which is the preferred choice in some parts of the world. The main concern of ACDF with stand-alone cages is the occurrence of subsidence; we have demonstrated this to be very low (0.15%) in our own population. Moreover, it has been proven that the different fusion techniques are equally safe. In fact, current literature challenges the use of plate constructs [94]. As described in the introduction and outlined in Table 1, most studies show lower rates of adjacent segment surgery in the ACDA groups. Hence, the goal for which the cervical disc prosthesis was designed appears to be (partly) reached. However, not all aspects have been sufficiently investigated. Only few studies have investigated the cost-effectiveness between techniques, although not in such an extensive, prospective, randomized controlled trial [41, 95].

External validity and generalizability are the main constraint of this study, as costs and preferred surgical technique are country-specific. For example, the costs of absenteeism are dependent on country, different salaries, and duration. The costs of surgery and hospital admission can also differ between countries, as can insurance reimbursement for ACDA. ACDA is not reimbursed in the Netherlands, while in other countries it is, and is offered as standard care. Nevertheless, we expect that the proportion of costs and effectiveness can be extrapolated to other countries, even though exact costs cannot. Importantly, the methodology of this study is reproducible and may encourage researchers around the world to also investigate cost-effectiveness in other settings. The inclusion of patients with single- and multi-level pathology and radiculopathy and/or myelopathy will provide an adequate representation of the daily practice, which ameliorates generalizability. Another limitation of this study is that the sample size is based on estimated costs and incidences of CASP, which may differ from reality.

A primary outcome based on different parameters, costs, and effects/utility remains relatively uncommon despite the increasing number of economic evaluations. No similar studies are available to estimate effect sizes; therefore, the sample size in this study might be over- or underestimated. There are no previous prospective trials on cost-effectiveness for ACDA versus ACDF, which does not allow for comparison.

If our hypothesis is not confirmed and this study shows that ACDF is more cost-effective than ACDA, there will be no change in the current standard treatment for CDDD. This study will remain valuable, as it will provide new insights into the existing debate concerning anterior decompression techniques in the treatment of CDDD.

If our hypothesis is proven correct and the chance of developing CASP is lower in ACDA than ACDF, ACDA has potential to lower direct and indirect healthcare costs. The gap in incidence of additional CASP-related surgery between ACDF and ACDA expands considerably with time (Table 1). We therefore expect that if a difference in costs within 4 years is detected, this difference will increase over time.

The Dutch National Society of Neurosurgeons supports the intentions of the study. The first step is to revise the national guidelines if the proposed study shows greater cost-effectiveness of ACDA compared to ACDF with equal clinical effectiveness. The ACDA procedure can then be implemented, leading to standard reimbursement. The process evaluation in this study will offer valuable insight into the optimal process surrounding ACDA.

### Dissemination policy

The findings of this study will be disseminated in conferences and seminars and will be published in an open-access international peer-reviewed journal.

## Trial status

Recruiting, started 01.01.2022. Anticipated end of study 01.06.2028.

## Supplementary Information

**Additional file 1.** SPIRIT figure CACES.

## Data Availability

The datasets generated during and/or analyzed during the current study are available from the corresponding author on reasonable request.

## Abbreviations

ACD: Anterior cervical discectomy
ACDA: Anterior cervical discectomy with arthroplasty
ACDF: Anterior cervical discectomy and fusion
ASP: Adjacent segment pathology
AE: Adverse events
AR: Adverse reaction
AVG: Algemene Verordening Gegevensbescherming
CACES: Cervical Arthroplasty Cost Effectiveness Study
CareQoL: Care related Quality of Life
CASP: Clinical adjacent segment pathology
CDDD: Cervical degenerative disc disease
CEA: Cost-effectiveness analysis
CUA: Cost-utility analysis
DCM: Degenerative cervical myelopathy
GCP: Good Clinical Practice
GDPR: General Data Protection Regulation (in Dutch: Algemene Verorderning Gegevensbescherming (AVG))
HADS: Hospital Anxiety and Depression Scale
IC: Informed consent
ICG: Informal caregiver
ICER: Incremental cost-effectiveness ratio
iMCQ: Medical Consumption Questionnaire
IMP: Investigational Medicinal Product
IMPD: Investigational Medicinal Product Dossier
iPCQ: Productivity Cost Questionnaire
iVICQ: Limited Valuation Of Informal Care Questionnaire
METC: Medical research ethics committee (MREC); in Dutch: medisch-ethische toetsingscommissie
MRI: Magnetic resonance imaging
NDI: Neck Disability Index
NMB: Net monetary benefit
NSAID: Non-steroid anti-inflammatory drug
PEEK: Polyether ether ketone
PROMs: Patient-reported outcome measures
QALY: Quality-adjusted life year
RASP: Radiological Adjacent Segment Pathology
SAE: Serious adverse event
SRB: Self-Related Burden Scale
VAS: Visual Analogue Scale
WTP: Willingness to pay
