# Supplementary material for: Research protocol: Cervical Arthroplasty Cost Effectiveness Study (CACES): economic evaluation of anterior cervical discectomy with arthroplasty (ACDA) versus anterior cervical discectomy with fusion (ACDF) in the surgical treatment of cervical degenerative disc disease — a randomized controlled trial
Source: Trials. 2022 Aug 26;23:715. doi: 10.1186/s13063-022-06574-5 (PMC9419384; doi:10.1186/s13063-022-06574-5)
Supplement: Supplementary file 1 — Additional file 1. SPIRIT figure CACES. [file 13063_2022_6574_MOESM1_ESM.doc]

SPIRIT Figure CACES

|  |  |  | | **STUDY PERIOD** | | | | | | | | |
| --- | --- | --- | --- | --- | --- | --- | --- | --- | --- | --- | --- | --- |
|  | **Enrolment** | | **Allocation** | |  |  | **Post-allocation** | | | | | **Close-out** |
| **TIMEPOINT**** | ***-t_1_*** | | **0** | | ***t_1_*** | ***t_2_*** | ***t_3_*** | ***T_4_*** | ***T5*** | ***T6*** | ***T7*** | ***T_8_*** |
| **ENROLMENT:** |  | |  | |  |  |  |  |  |  |  |  |
| **Eligibility screen** | X | |  | |  |  |  |  |  |  |  |  |
| **Informed consent** | X | |  | |  |  |  |  |  |  |  |  |
| **Register trial participation in EPD** | X | |  | |  |  |  |  |  |  |  |  |
| **Pre-operative questionnaires** | X | |  | |  |  |  |  |  |  |  |  |
| **Pre-operative imaging** | X | |  | |  |  |  |  |  |  |  |  |
| **Inform GP of study participation** |  | |  | |  |  |  |  |  |  |  |  |
| **Allocation** |  | | X | |  |  |  |  |  |  |  |  |
| **INTERVENTIONS** |  | |  | |  |  |  |  |  |  |  |  |
| ***ACDA*** |  | |  | | X | X | X | X | X | X | X | X |
| ***ACDF*** |  | |  | | X | X | X | X | X | X | X | X |
| **ASSESSMENTS:** |  | |  | |  |  |  |  |  |  |  |  |
| ***Questionnaires*** |  | |  | |  | X | X | X | X | X | X | X |
| ***Radiologic outcomes*** |  | |  | |  | X |  |  |  |  |  | X |

*Recommended content can be displayed using various schematic formats. See SPIRIT 2013 Explanation and Elaboration for examples from protocols.

**List specific timepoints in this row.
